# Optimized coil arrangement for integrating leakage inductance in high-frequency transformers of dual active bridge converters

**DOI:** 10.1038/s41598-025-19533-2

**Published:** 2025-10-13

**Authors:** Kianoush Yadollahi, Adel Zakipour

**Affiliations:** Department of Electrical and Computer Engineering, Arak University of Technology, Arak, Iran

**Keywords:** Dual active bridge (DAB) power converter, Integrated leakage inductance design, Transformer winding optimization, Coil arrangement, Finite element electromagnetic analysis, Electrical and electronic engineering, Energy storage

## Abstract

Dual Active Bridge (DAB) converters offer bidirectional power flow and soft-switching capabilities, making them attractive for high-frequency power conversion applications. However, the presence of a separate series inductor alongside the high-frequency transformer can limit the converter’s power density and efficiency. This paper presents an optimized transformer winding configuration that integrates the required leakage inductance directly within the high-frequency transformer, eliminating the need for an external series inductor. Various coil arrangements are analyzed through finite element simulations using ANSYS Maxwell to evaluate leakage inductance, ohmic losses, and parasitic capacitance. Results show that the vertical coil arrangement achieves the target leakage inductance of 50 μH while minimizing conduction losses and parasitic effects. The optimized transformer winding was experimentally validated on a 150 W DAB converter prototype using a TMS320F28335 DSP for power stage control. Experimental results confirm close agreement with simulations, demonstrating high efficiency (up to 99.1%) and stable soft-switching operation under nominal conditions without current ringing. The proposed winding configuration offers a practical and efficient approach for integrating magnetic components in high-frequency power converters.

## Introduction

As global energy demand continues to rise, the development of electric vehicles (EVs) has gained significant momentum due to their environmental advantages. Consequently, fast and compact DC-DC converters are becoming increasingly important for charging infrastructure. Among various topologies, Dual Active Bridge (DAB) converters have become a favorable solution due to their galvanic isolation, high power density, soft-switching capability, and bidirectional power flow^1,2^.

To meet the stringent performance requirements of modern power systems, researchers have explored advanced control and modulation strategies to improve the switching behavior of DAB converters. Techniques such as Zero Voltage Switching (ZVS) and Zero Current Switching (ZCS) help reduce switching losses and thermal stress on semiconductor devices^3–6^. Furthermore, modulation methods such as Dual Phase Shift (DPS), Extended Phase Shift (EPS), and Triple Phase Shift (TPS) have been introduced to extend the soft-switching range and limit reactive power flow^7–12^.

Despite these advancements, magnetic components like inductors and transformers contribute to relatively high magnetic and ohmic losses in converters, consequently decreasing their efficiency and power density. Thus, introducing effective methods to reduce losses caused by magnetic elements can improve the overall performance of converters. As a fundamental solution, the series inductor with the high-frequency transformer can be eliminated and integrated as leakage inductance within the transformer using different coil arrangements. Leakage inductance in transformers can be achieved either by an air gap or by adding a magnetic core with suitable relative permeability instead of the air gap^13–16^; however, this approach leads to increased core losses, larger dimensions, and EMI noise^17^. The proposed method for generating leakage inductance within the high-frequency transformer involves creating a gap between the primary and secondary windings through their vertical or horizontal arrangement. Although the concept of integrating leakage inductance within the high-frequency transformer of DAB converters has been explored to some extent, in many previous studies, such as^18^, series inductors have been used alongside the transformer to implement the converter. While some studies^19–22^ have investigated the integration of leakage inductance using finite element analysis (FEM) with the arrangement of transformer windings horizontally and vertically. These references do not provide a comprehensive analysis of all the characteristics affecting transformer design including core losses, winding losses, winding resistance, leakage inductance and magnetizing inductance. Instead, the main approach of these references has been to only combine the leakage inductance with the transformer. In^23^, the leakage inductance for three DAB converters in parallel with a power of 1.2 kW and a frequency of 1 MHz is integrated in the transformer. In this structure, three coils are placed on a core, and an analysis including the calculation of coil losses is also provided.

In^24^, the leakage inductance of the high-frequency transformer in the DAB converter was modelled using the energy law for litz wire coils with horizontal arrangements, and leakage energy distribution results along with magnetic field intensity were extracted using the FEM method. Similarly, in^25^, leakage inductance was modelled using the energy law, and a wide range of leakage inductance values was produced by utilizing horizontal coil arrangements and introducing an air gap in the transformer structure. In^26^, horizontal and vertical coil structures were placed within a U-shaped transformer core in the DAB converter, and optimal transformer design was performed considering magnetic losses and minimizing leakage inductance. The transformer’s performance was also evaluated for optimal insulation quality using FEM analysis. In^27^, leakage inductance modelling for different coil arrangements was conducted based on the reluctance path calculation method for a planar transformer in the DAB converter, and the magnetic flux vector results were extracted using FEM analysis. In^28^, leakage inductance was integrated within transformers using various coil arrangements while considering the ohmic losses of windings, and its validation was carried out through FEM analysis. The^29^ demonstrates interleaved and non-interleaved structures for integrating leakage inductance within Core Type and Shell Type high-frequency transformers in DAB converters were analysed and investigated. Additionally, a comprehensive flowchart for optimal transformer design considering core and copper losses was presented.

While several studies have utilized different coil arrangements to integrate leakage inductance within transformers, a comprehensive analysis including ohmic losses of windings, core losses, and the parasitic capacitance generated by these structures has not been provided. However, despite the above studies, the existing literature lacks an in-depth, quantitative, and comparative approach that evaluates all critical transformer parameters across different winding configurations and provides clear design guidelines. The main innovations of this paper, addressing these research gaps, are summarized as follows:

1. Comprehensive investigation of how different winding arrangements affect leakage inductance, using FEM analysis.
2. Calculation of core losses, winding losses, and winding resistance for each proposed structure.
3. Assessment of leakage capacitance in various proposed arrangements.

Table 1 presents a comprehensive comparison between our study and key referenced works, highlighting the broader and more detailed scope of our analysis.

**Table 1:** presents a comparison between the proposed method and existing references.

Measuremet parameters	Leakage inductance	Magnetizing inductance	Winding resistance	Winding loss	Core loss	Winding capacitance	Winding arrangement type analysis	FEM simulation
Proposed							Horizontal and vertical	
Ref.^19^]							Horizontal and vertical	
Ref.^20^							Horizontal	
Ref.^21^							Vertical	
Ref.^22^							Vertical	
Ref.^23^							-	

In this study, an integrated transformer design methodology is proposed, wherein the required series inductance of a Dual Active Bridge (DAB) converter is embedded as controlled leakage inductance within the high-frequency transformer through optimized winding geometry. Four distinct coil configurations are investigated using finite element analysis (FEA) in ANSYS Maxwell, assessing critical parameters including leakage and magnetizing inductances, copper losses, core losses, and interwinding capacitance. Among the evaluated topologies, the vertical winding arrangement demonstrates superior performance, achieving the target leakage inductance of 50 μH while minimizing parasitic effects and conduction losses. To validate the simulation results, a 150 W hardware prototype of the DAB converter is implemented, confirming the feasibility and effectiveness of the proposed transformer configuration through experimental measurements.

The structure of this paper reflects the progressive development of the proposed transformer design. Section "DAB and integration leakage inductance theory" introduces the operating principles of the DAB converter and formulates the theoretical framework for embedding leakage inductance through controlled winding geometry. Section "Electromagnetic performance" details the finite element modeling approach and presents a comparative evaluation of four winding configurations in terms of electromagnetic and parasitic performance. Section "Experimental results" describes the experimental implementation and measurement methodology used to validate the simulation outcomes. Section "Conclusion" concludes the paper by summarizing the core findings and outlining the implications for high-frequency transformer design in advanced power conversion systems.

## DAB and integration leakage inductance theory

Leakage inductance is a key parameter influencing the operational efficiency and dynamic behavior of Dual Active Bridge (DAB) converters. This section develops a comprehensive modeling framework for the purposeful integration of leakage inductance within the high-frequency transformer design, emphasizing its impact on converter performance.

### DAB converter

**Fig. 1:**
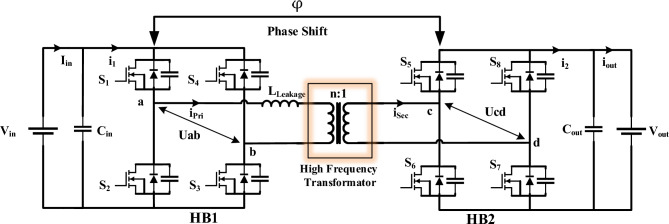
Fundamental configuration of a Dual Active Bridge (DAB) converter.

The general structure of the DAB converter consists of two H-Bridges, a high-frequency transformer, and a series inductor with it, as shown in Fig. 1.

The output power of the converter depends on the series inductor with the transformer, the phase shift between the two H-Bridges, the input and output voltages, the transformer turns ratio, and the switching frequency, which is expressed by Eq. 1.

1 $${P}_{Out}=\frac{n{V}_{in}{V}_{Out}}{2{f}_{s}.{L}_{Leakage}}\varphi \left(1-\varphi \right)$$

where ${P}_{Out}$ is the output power, ${V}_{in}$ is the input voltage of the primary H-Bridge, ${V}_{Out}$ is the output voltage of the secondary H-Bridge, ${f}_{s}$ is switching frequency, ${L}_{Leakage}$ is leakage inductance referred to the primary winding of the transformer, $n$ is the transformer ratio and $\varphi$ is the phase shift between two H-Bridges, respectively.

### Integration leakage inductance theory

A major portion of energy in the DAB converter is transferred through the series inductor paired with its transformer. Therefore, transferring this energy while minimizing losses and improving efficiency is of utmost importance.

To reduce losses caused by magnetic components and decrease the size of the DAB converter, the series inductor with the high-frequency transformer can be eliminated, and energy can be transferred using leakage inductance.

Various coil arrangements within the transformer window provide an effective method for generating controlled flux leakage and, consequently, leakage inductance within the transformer windings. This approach enables the direct integration of leakage inductance into the transformer structure.

Considering that the leakage inductance value depends on core dimensions, spacing between windings, and the number of turns, the initial transformer design plays a fundamental role in estimating the achievable leakage inductance. Established design methodologies for high-frequency transformers are thoroughly reviewed in^30–36^. In this study, the initial transformer design follows the methodology presented in^35^, utilizing an ER-shaped ferrite core composed of MnZn material, which is suitable for operating frequencies between 20 and 100 kHz. This core offers key features such as a relatively high inductance-per-turn ratio, a rounded center leg, and manufacturability advantages including ease of winding. The detailed specifications of the designed high-frequency transformer are summarized in Table 2.

**Table 2:** The main parameters of the designed high-frequency transformer*.*

Parameters	Value	Unit
Effective magnetic cross section (A_e_)	1.7	(Cm^2^)
Effective magnetic path length (l_e_)	9.9	(Cm)
Effective magnetic volume (V_e_)	16.8	(Cm^3^)
Mean length per turn (MLT)	4.7123	(Cm)
Area product (A_P_)	2.04204	(Cm^4^)
Effective window area (W_a_)	1.2012	(Cm^2^)
Maximum flux density (B_Max_)	0.153	Tesla
Primary winding turns (N_P_)	28	Turn
Secondary winding turns(N_s_)	14	Turn
Primary winding cross section (A_WP_)	0.005362	(Cm^2^)
Secondary winding cross section (A_WS_)	0.010725	(Cm^2^)

**Fig. 2:**
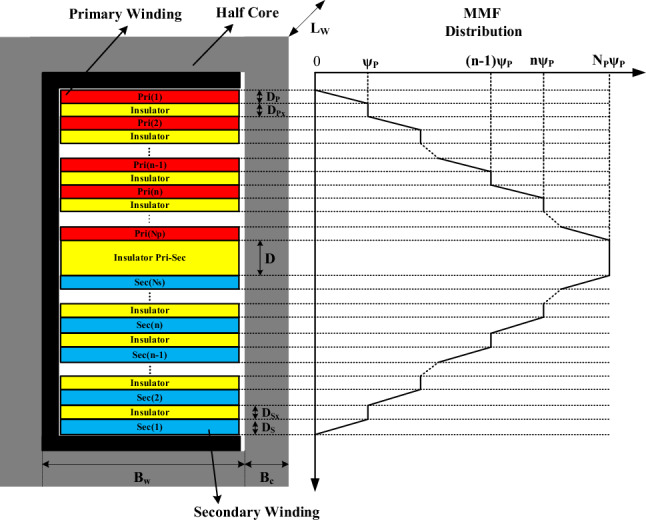
MMF diagram of vertical arrangement of windings in half of the transformer.

Vertical coil arrangement compared to horizontal arrangement leads to greater weakening of the magnetic coupling coefficient and produces higher flux and leakage inductance in the transformer. Therefore, vertical coil arrangement is utilized to achieve the desired leakage inductance in the high-frequency transformer of the DAB converter. In this regard, the comprehensive and general layout of the coil arrangement within half of the high-frequency transformer’s window, layer by layer, along with the use of insulation between them, is very helpful in plotting the MMF diagram based on the location of layers (x) and calculating the magnetic field intensity of the targeted layer. This structure is illustrated in Fig. 2.

On the left side of Fig. 2, the layer of the primary winding is labelled $Pri(n)$ with a width of ${D}_{p}$, and the layer of the secondary winding is labeled $Sec(n)$ with a width of ${D}_{s}$. The number of turns in each layer of the primary winding is denoted by ${N}_{Lp}$, and the number of turns in each layer of the secondary winding is denoted by ${N}_{Ls}$. Ultimately, the ampere-turns of the nth layer of the primary winding are represented as ${N}_{Lp}{I}_{p}$, and the ampere-turns of the nth layer of the secondary winding are represented as ${N}_{Ls}{I}_{s}$. Additionally, between each layer of the primary winding, there is an insulator with a width of ${D}_{Px},$ between each layer of the secondary winding, an insulator with a width of ${D}_{Sx}$, and between the two windings, an insulator with a width of $D$ is placed.

**Fig. 3:**
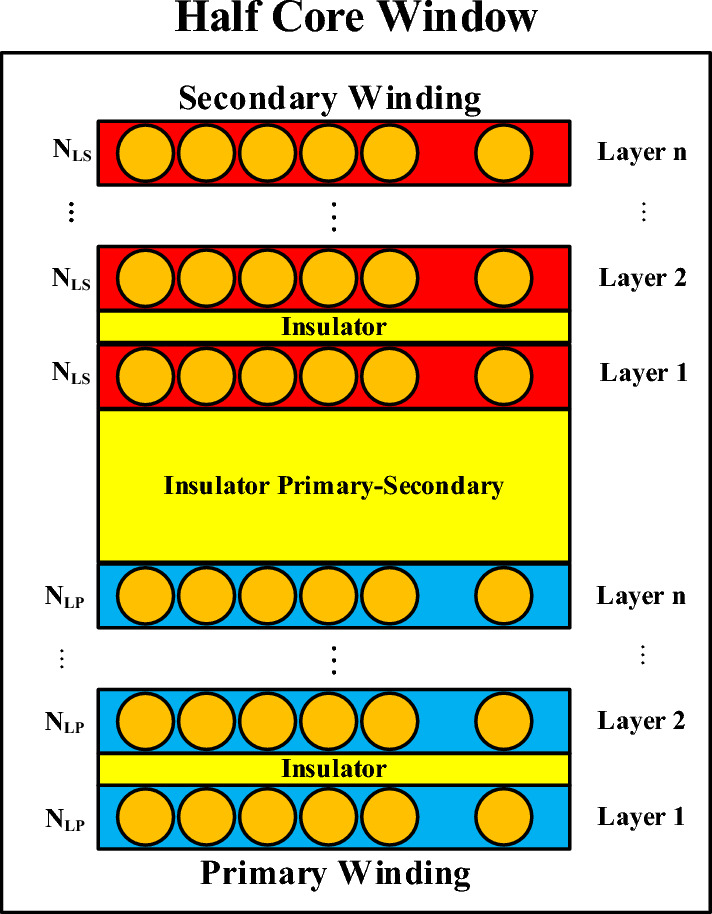
distribution of primary and secondary windings in the transformer.

On the right side, the MMF distribution diagram of the layers within half of the transformer’s window is illustrated, where the ampere-turns of the first layer of the primary winding are indicated by $\uppsi {}_{P}$. The components within the transformer window are depicted in more detail in Fig. 3. Each of the primary and secondary windings consists of ${N}_{P}$ and ${N}_{s}$ layers, respectively, shown in blue and red, with the number of turns per layer represented by ${N}_{Lp}$ and ${N}_{Ls}$, respectively. Moreover, yellow insulators are placed between the layers and the two windings.

**Fig. 4:**
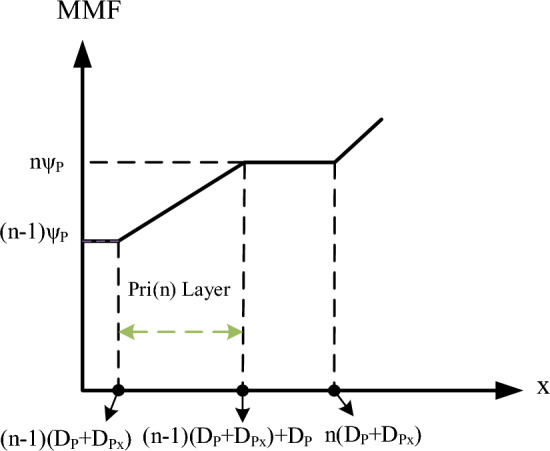
MMF distribution diagram of primary windings.

To calculate leakage inductance, the stored energy in the primary and secondary windings must be obtained. In this regard, the MMF distribution diagram based on the location of the layers is helpful for calculating the stored energy in the primary ${E}_{pri}$ or secondary ${E}_{sec}$ windings. In Fig. 4, the MMF distribution diagram of the transformer’s primary windings are illustrated.

Based on the MMF distribution diagram presented in Fig. 4, the magnetic field intensity as a function of the winding position is provided in Eq. (2). In the first range, the variations in magnetic field intensity occur between points A and B, while in the second range, these variations occur between points B and C. Therefore, using the energy law, the energy stored in the primary winding is calculated according to Eq. (3). By simplifying Eq. (3), the stored energy in the primary winding is obtained according to Eq. (4). Similarly, the energy stored in the secondary winding is calculated according to Eq. (5). Additionally, for the insulator placed between the primary and secondary windings, Eq. (6) can be derived. This equation represents the energy flowing from the primary and secondary windings towards the insulator between them. Thus, the leakage inductances of the primary and secondary windings are denoted as ${L}_{KP}$ and ${L}_{Ks}$, respectively, and are expressed in Eq. (7). Finally, the leakage inductance referred to the primary side of the transformer is calculated according to Eq. (8).

**Fig. 5:**
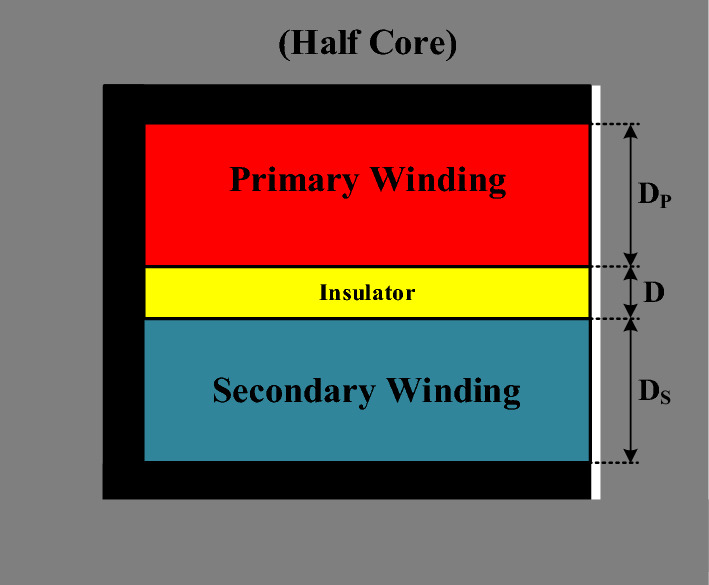
Simplified structure of winding arrangement inside the transformer.

According to Fig. 5, for simplifying the equations and considering a realistic arrangement, the values of $Dsx$ and $Dpx$ are set to zero, and only the insulator between the windings (with a width of $D$) is considered as the main contributor to the production of leakage inductance. Additionally, it is assumed that the primary winding has 28 turns in one layer and the secondary winding has 14 turns in another layer.

Therefore, for the new structure, Eqs. (3) to (7) can be simplified, leading to Eq. (9) for calculating the leakage inductance of the configuration illustrated in Fig. 5.

$$H\left(x\right)=\left\{\genfrac{}{}{0pt}{}{\frac{\left(n-1\right){\psi }_{P}}{{B}_{w}}+\frac{{\psi }_{p}}{{B}_{w}{D}_{P}}\left(x-\left(n-1\right)\left({D}_{P}+{D}_{Px}\right)\right)}{\left(n-1\right)\left({D}_{P}+{D}_{Px}\right)<x\le \left(n-1\right)\left({D}_{P}+{D}_{Px}\right)+{D}_{P}}\right\}$$

2 $$H\left(x\right)=\left\{\genfrac{}{}{0pt}{}{\frac{n{\psi }_{P}}{{B}_{w}}}{\left(n-1\right)\left({D}_{P}+{D}_{Px}\right)+{D}_{P}<x\le n\left({D}_{P}+{D}_{Px}\right)}\right\}$$

3 $${E}_{pri}={\mu }_{0}{L}_{w}{B}_{w}\left\{\sum_{n=1}^{{N}_{P}}{\int }_{\left(n-1\right)\left({D}_{P}+{D}_{Px}\right)}^{\left(n-1\right)\left({D}_{P}+{D}_{Px}\right)+{h}_{p}}{H}^{2}\left(x\right)dx+\sum_{n=1}^{{N}_{P}-1}{\int }_{\left(n-1\right)\left({D}_{P}+{D}_{Px}\right)+{D}_{P}}^{n\left({D}_{P}+{D}_{Px}\right)}{H}^{2}\left(x\right)dx\right\}$$

4 $${E}_{pri}=\frac{1}{6}{\mu }_{0}\frac{{L}_{w}}{{B}_{w}}{N}_{Lp}^{2}\left[2{D}_{P}{N}_{P}^{3}+{D}_{Px}(2{N}_{P}^{3}-3{N}_{P}^{2}+{N}_{P})\right]{I}_{P}^{2}$$

5 $${E}_{Sec}=\frac{1}{6}{\mu }_{0}\frac{{L}_{w}}{{B}_{w}}{N}_{Ls}^{2}\left[2{D}_{S}{N}_{S}^{3}+{D}_{Sx}(2{N}_{S}^{3}-3{N}_{S}^{2}+{N}_{S})\right]{I}_{S}^{2}$$

6 $${E}_{in{s}_{P},S}={\mu }_{0}\frac{D{L}_{w}}{{B}_{w}}{N}_{Lp,S}^{2}{N}_{P,S}^{2}{I}_{P,S}^{2}$$

$${L}_{KP}=\frac{2({E}_{pri}+{E}_{in{s}_{P}})}{{I}_{P}^{2}}$$

7 $${L}_{KS}=\frac{2({E}_{Sec}+{E}_{in{s}_{S}})}{{I}_{S}^{2}}$$

8 $${L}_{Leakage}={L}_{KP}+{\left(\frac{{N}_{P}\times {N}_{Lp}}{{N}_{S}\times {N}_{Ls}}\right)}^{2}{L}_{KS}$$

9 $${L}_{Leakage}=2{\mu }_{0}\frac{{L}_{W}}{{B}_{W}}{N}_{LP}^{2}[\frac{1}{3}\left({D}_{P}+{D}_{S}\right)+2D]$$

**Fig. 6:**
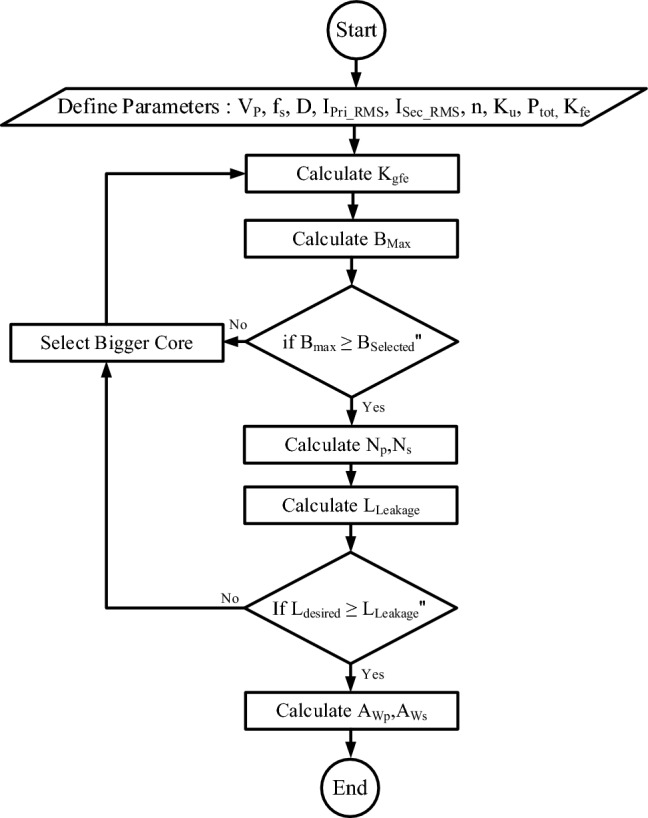
Flowchart of integrating leakage inductance into a high-frequency transformer.

Using Eq. (9), the leakage inductance can be adjusted to the desired value by tuning the parameters $D$, ${D}_{p}$, and ${D}_{s}.$ The dimensions required for integrating a leakage inductance of 50 microhenries within the high-frequency transformer are presented in Table 3. As a general conclusion, the flowchart for integrating leakage inductance within the high-frequency transformer can be illustrated as shown in Fig. 6. In this flowchart, ${V}_{p}$ represents the maximum voltage applied to the transformer, and $D$ indicates the duty cycle.

**Table 3:** Geometrical and electrical parameters of transformer core and windings.

Parameters	Value	Unit
Primary winding height (D_P_)	14.9	(mm)
Secondary winding height (D_S_)	14.9	(mm)
Primary-secondary insulator height (D)	1.38	(mm)
L_W_	15	(mm)
B_C_	5.75	(mm)
B_W_	7.5	(mm)
Leakage inductance (referred primary)	50.021	(uH)

## Electromagnetic performance

In this section, numerical software was used to analyse what was stated previously. The reference value of ${K}_{gfe}$ obtained value for the designed high-frequency transformer using the method presented in^29^ is 0.0143. The ER/35/21/11 magnetic core, with a ${K}_{gfe}$ greater than the reference value is suitable for energy transfer. However, due to the small height of this core, it is not possible to achieve the desired leakage inductance by adjusting the gap between the windings, and the maximum achievable leakage inductance is approximately 28 µH. This issue can be analysed using Eq. (9). According to the flowchart presented in Fig. 6, the ER/42/22/15 core with ${K}_{gfe}$ = 0.0250, offering a higher range than the previous core, can be used to achieve a leakage inductance of up to 120 µH. The physical specifications of this core are accessible in Table 2. The various coil arrangements within the high-frequency transformer will be simulated and analysed using Ansys Maxwell software in the Eddy Current environment, with the frequency set to 20 kHz. Values for leakage inductance, magnetizing inductance referred to the primary winding, winding ohmic resistance, winding losses, and core losses will be obtained.

Additionally, to analyse the parasitic capacitance of the windings, the design will be transferred to the Electrostatic environment, and the desired results will be extracted.

**Fig. 7:**
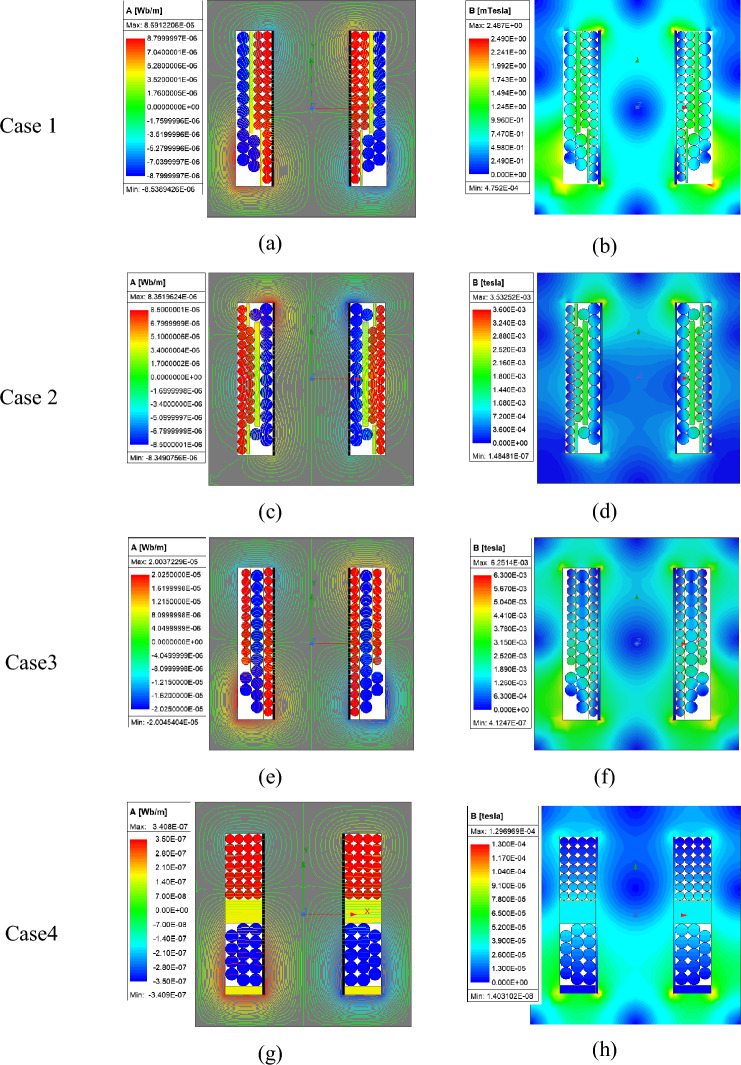
Different arrangements of windings inside the transformer window for Cases (1–4).

In this section, different winding arrangements will be simulated and analysed in Cases 1–4, and the most suitable configuration, with minimal ohmic losses, lowest parasitic capacitance, and desired leakage inductance, will be selected. The results of magnetic flux density and flux lines for the four cases are shown on the right and left sides of Fig. 7, respectively. The primary windings are represented in red, while the secondary windings are shown in blue, placed within the transformer window.

In Cases (1–3), due to strong magnetic coupling between the windings, low leakage inductance is generated, and most of the leakage flux is observed in parts of the core due to the gap between the windings, resulting in ohmic losses in the windings. In Case (4), the primary and secondary windings are positioned at the top and bottom of the magnetic core, respectively, with yellow insulation between them. In this configuration, by adjusting the gap between the windings and weakening the magnetic coupling between them, leakage flux around the windings can be increased to achieve high leakage inductance. Compared to the previous configurations, this structure exhibits a larger range of leakage inductance, significantly lower ohmic losses, and reduced parasitic capacitance.

**Fig. 8:**
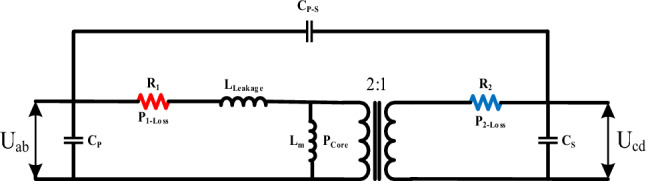
Electrical equivalent circuit (manipulated) of a high-frequency transformer.

The parametric values of magnetizing inductance, leakage inductance, winding resistance, winding losses, core losses, and parasitic capacitance in the equivalent electrical circuit of the high-frequency transformer are depicted in Fig. 8. The simulation results for these parameters across the 4 cases are summarized in Table 4.

**Table 4:** Simulation results of different cases.

	Cp,Cs,Cp-s	PCore	P1-Loss, P2-Loss	R1, R2	Lm	LLeakage
Case (1)	Cp = 665 pF Cs = 669 pF Cp-s = 656 pF	97.5 mW	P1-Loss = 0.93 W P2-Loss = 1.91 W	$1m\Omega$	4.75 mH	4.175 uH
Case (2)	Cp = 545 pF Cs = 538 pF Cp-s = 536 pF	35 mW	P1-Loss = 1.36 W P2-Loss = 2.8 W	$1m\Omega$	4.75 mH	3.43 uH
Case (3)	Cp = 2000 pF Cs = 2000 pF Cp-s = 2000 pF	36 mW	P1-Loss = 3.25 W P2-Loss = 4.62 W	$1m\Omega$	4.75 mH	2.67 uH
Case (4) Proposed	Cp = 277 pF Cs = 277 pF Cp-s = 267 pF	27 mW	P1-Loss = 0.06 W P2-Loss = 0.013 W	$1m\Omega$	4.75 mH	50.024 uH

According to Table 4, the values of magnetizing inductance and the ohmic resistance of the windings remain unchanged as they depend on the dimensions and material properties. Moreover, the proposed structure demonstrates significantly lower values of parasitic capacitance, ohmic losses, and core losses compared to other configurations. Therefore, this arrangement not only achieves the desired leakage inductance but also incorporates these advantageous features.

**Fig. 9:**
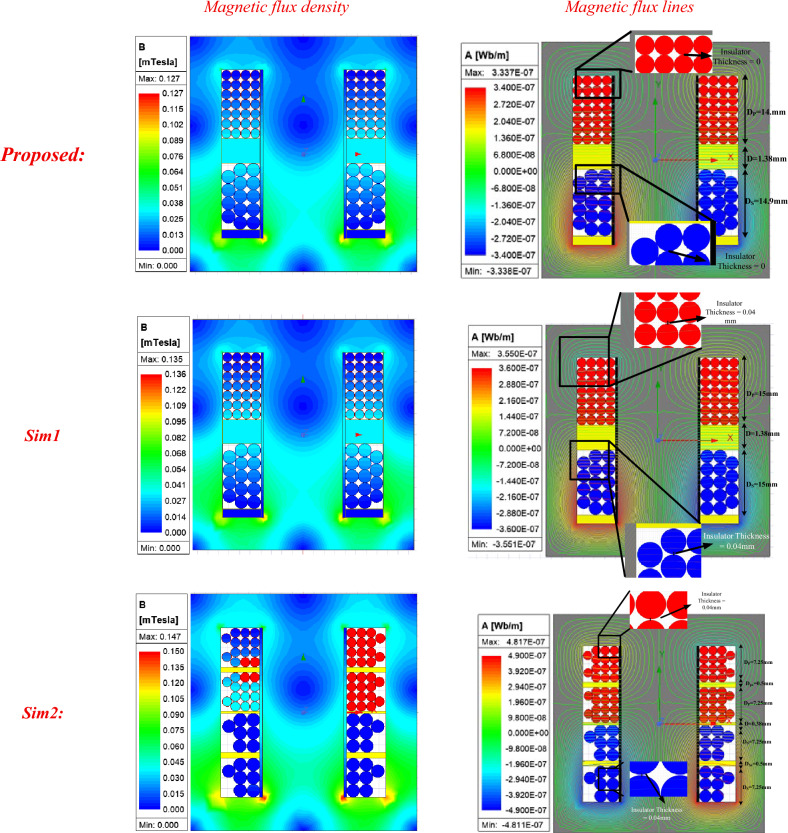
simulation results of the effect of Dpx on the leakage inductance considering with and without wire insulation.

In addition, the influence of the interlayer insulation thicknesses (*Dsx* and *Dpx*) on the leakage inductance was evaluated in accordance with the IEC 60,317 standard, which specifies a nominal enamel coating thickness of 0.02–0.06 mm for round copper conductors. The simulation results indicate that incorporating this enamel thickness (0.04 mm in our analysis) causes a negligible change in *L*_*Leakage*_ compared with the proposed configuration without enamel. By contrast, a significant change in *L*_*Leakage*_ occurred only when the main insulation distance between the primary and secondary windings (*D*) was reduced -for example, from 1.38 mm to 0.38 mm- which yielded a substantial decrease in leakage inductance. While such a reduction of *D* is impractical in real transformer designs due to insulation and mechanical constraints, it was included here to illustrate the high sensitivity of leakage inductance to this parameter, as summarized in Table 5 and depicted in Fig. 9.

**Table 5:** Leakage inductance changes for different insulator distances in existing simulations (all of the dimensions are in millimeters).

	NP	NLP	NS	NLS	DPx	DP	DSx	DS	D	Llk_sim
Proposed (without wire insulator thickness)	1	28	1	14	-	14.9	-	14.9	1.38	50.02
Sim1 (with considering wire insulator thickness)	1	28	1	14	-	15	-	15	1.38	50.021
Sim2 (with considering wire Insulator thickness and interlayer insulator)	2	14	2	8	0.5	7.25	0.5	7.25	0.38	39.76

**Fig. 10:**
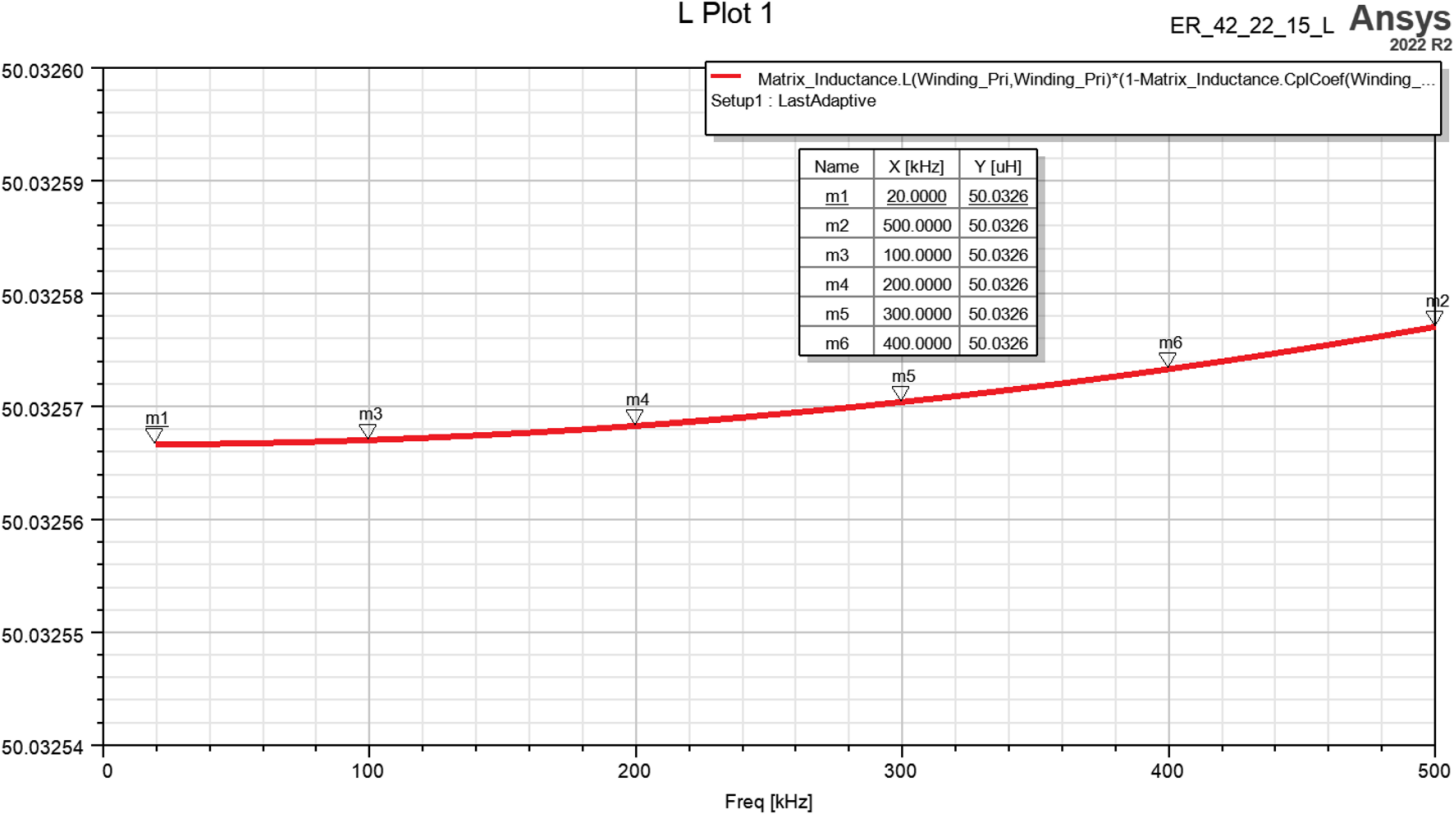
Results of leakage inductance variation versus frequency.

To further investigate the frequency dependence of the leakage inductance in the proposed high-frequency transformer, a frequency sweep analysis was performed across the range of 40 to 500 kHz, as illustrated in Fig. 10. The results indicate that the leakage inductance remains essentially constant throughout this frequency range, with observed variations being less than 0.2%. This demonstrates that increasing the operating frequency has a negligible effect on the leakage inductance.

On the other hand, the influence of increasing frequency on the skin and proximity effects has also been analyzed. These phenomena primarily manifest as a rise in ohmic losses in transformer windings and core, while their impact on the actual leakage inductance value remains negligible. Table 6 presents a comparative analysis of average copper and core losses at four different switching frequencies, clearly showing that while AC losses in the windings increase with frequency, the leakage inductance remains essentially constant. According to Faraday’s law, applying a voltage excitation to the transformer leads to a decrease in magnetic flux density as frequency rises, which consequently lowers core losses in accordance with the relation ${P}_{core}=Kf{B}^{\beta }$.

**Table 6:** Results of winding and core losses at frequencies from 40 to 200 kHz.

	P_Core_	Pcu (Pri&.Sec.)	${\text{Pcu}}_{\text{Freq}}/{\text{Pcu}}_{40\text{KHz}}$
40kHz	32.358 mW	69.065 mW	100%
100kHz	10 mW	93.45 mW	135.3%
150kHz	5.16 mW	100.18 mW	145%
200kHz	2.076 mW	115.78 mW	167.63%

## Experimental results

**Fig. 11:**
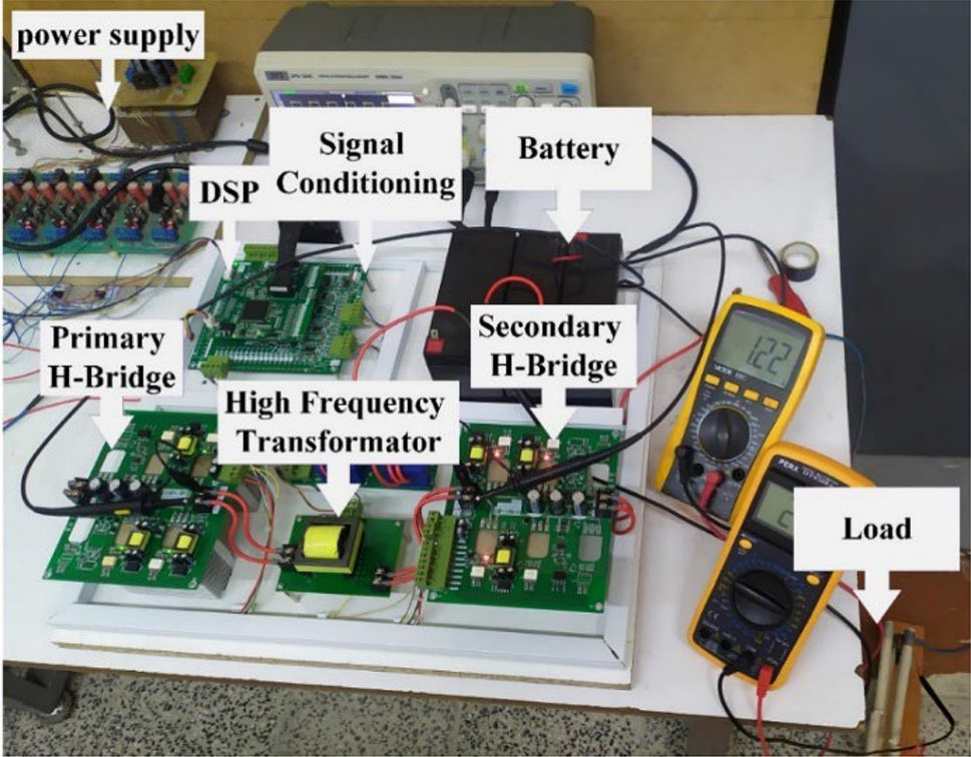
Test bench of the desired DAB.

After simulation and ensuring the desired leakage inductance is achieved, a practical prototype can be tested using an LCR meter. To evaluate the performance of the constructed transformer in the DAB converter, the experimental setup shown in Fig. 11 has been implemented. In this laboratory prototype, a DSP microprocessor with the specifications TMS320F28335 manufactured by TI is used to generate gate signals for the switches. The electrical parameters of the DAB converter are provided in Table 7. To measure the leakage inductance observed from the primary side, the secondary winding terminals can be short-circuited, and the probes of the LCR meter can be connected to the terminals of the primary winding. Additionally, to measure the magnetizing inductance, the secondary winding terminals can be left open-circuited. The parasitic capacitance between the primary and secondary windings can be calculated by individually short-circuiting the primary and secondary windings and connecting the remaining terminals to the probes of the LCR meter. The values obtained from practical measurements and simulations are presented in Table 8, showing minor differences between them.

**Table 7:** The main electrical parameters of DAB.

Parameters	Symbol	Value
Input voltage range	V_in_ (V)	40–60
Output voltage range	V_out_ (V)	24–28
Output power rating	P_Nom_ (W)	150
input current maximum	I_in_Max_ (A)	4
output current maximum	I_out_Max_ (A)	6
Transformer primary current (RMS)	I_Pri_RMS_ (A)	2.5
Transformer secondary current (RMS)	_ISec_RMS_ (A)	5
Efficiency	$\upeta (\text{\%})$	99% (full load)
Switching frequency	f_s_ (KHz)	40
Leakage inductance	L_Leakage_ (uH)	50
Transformer ratio	n	2

**Table 8:** A numerical comparison of simulation and experimental results.

	Parameters				
Results	L_m_	L_Leakage_	C_p-s_	R_1_	R_2_
Simulation result	4.75 mH	50.024 uH	267 pF	$1m\Omega$	$1{\varvec{m}}{\varvec{\Omega}}$
Experimental result	4.81 mH	50.01 uH	293 pF	1.48 $m\Omega$	1.26 ${\varvec{m}}{\varvec{\Omega}}$

**Fig. 12:**
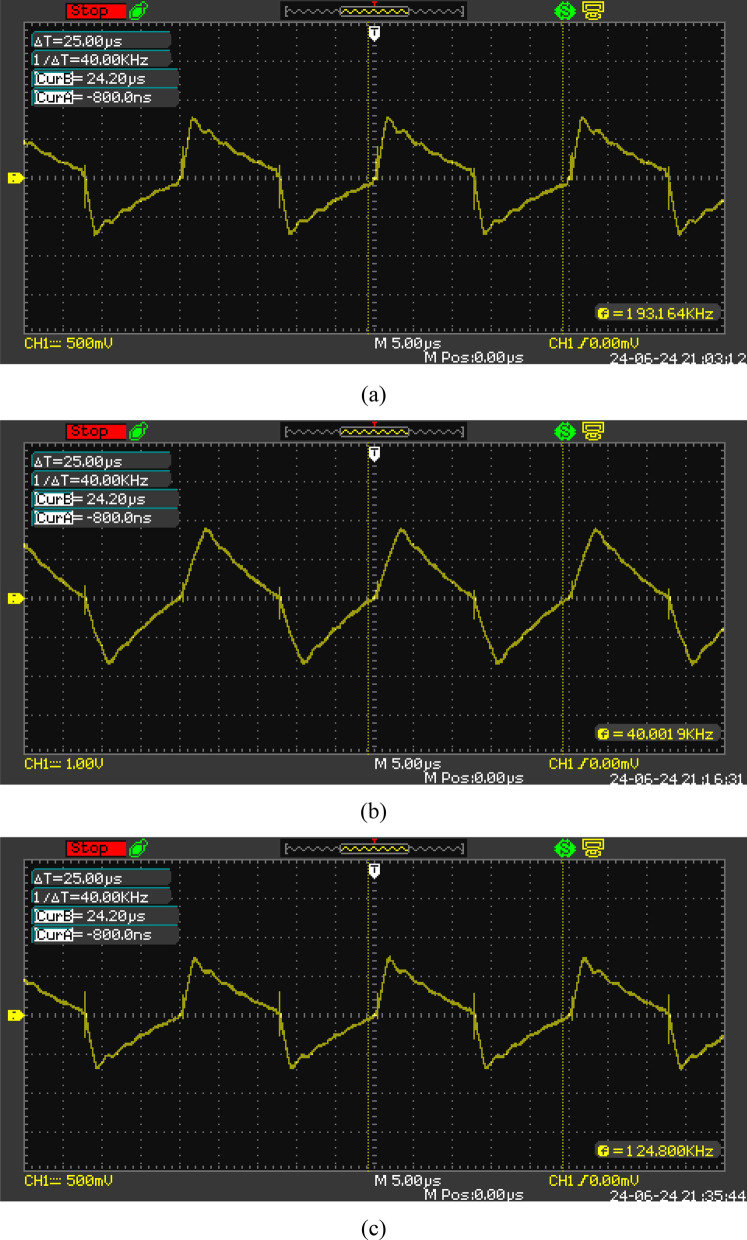
Leakage inductance current in the performed tests: (**a**) Leakage inductance current in Test 1 (vertical axis 0.77A/Div and horizontal axis 5us/Div), (**b**) Leakage inductance current in Test 2 (vertical axis 0.7A/Div and horizontal axis 5us/Div), (**c**) Leakage inductance current in Test 3 (vertical axis 0.77A/Div and horizontal axis 5us/Div).

The transformer’s performance in the DAB converter is evaluated through three practical tests under Single Phase Shift (SPS) modulation, and the leakage inductance current on the primary side of the transformer is measured. Table 9 presents the results of the different tests. Based on Fig. 12, it is evident from the first test that the converter has maintained its soft switching capability, resulting in high efficiency. However, in the second and third tests, the efficiency of the converter decreases due to the loss of the soft switching capability. Additionally, the achieved optimal parasitic capacitance values have prevented ringing in the leakage inductance current.

**Table 9:** A comparison of the obtained results from the practical test in different tests.

Test Number	V_in_	V_out_	I_in_	I_out_	PhaseShift (degree)	Efficiency (%)
Test (1)	48	26	1.03	1.58	12.38	99.1
Test (2)	48	40.8	1.85	2.1	14.54	96.48
Test (3)	40	31	0.965	1.39	11.15	97.94

## Conclusion

In this paper, the series inductor with the high-frequency transformer of the DAB converter was removed to reduce the number of magnetic components. Following the modelling of the vertical coil arrangement, a flowchart for the transformer design process and leakage inductance integration was provided. Simulations of various coil arrangements within the transformer’s window were conducted to evaluate parameters such as magnetizing and leakage inductances, winding resistance and losses, core losses, and parasitic capacitance. The vertical arrangement was identified as the optimal configuration due to its minimal losses and parasitic capacitance. Furthermore, to verify the converter’s performance in the presence of the transformer, a laboratory setup of the DAB converter with 150 W power and a switching frequency of 40 kHz was implemented. The results of three tests revealed that the converter achieved an efficiency of 99.1% under nominal conditions. However, in other tests, the efficiency decreased due to changes in the voltage transformation ratio on both sides of the transformer and deviation from the defined soft-switching region. Additionally, the leakage inductance current in the tests exhibited no ringing due to the optimal parasitic capacitance achieved.

## Data Availability

All data generated or analyzed during this study are included in this article. However, all datasets used during the current study are also available from the corresponding author on reasonable request.
